# A *Saccharomyces* paradox: chromosomes from different species are incompatible because of anti-recombination, not because of differences in number or arrangement

**DOI:** 10.1007/s00294-019-01038-x

**Published:** 2019-11-20

**Authors:** Jasmine Ono, Duncan Greig

**Affiliations:** University College London, London, UK

**Keywords:** Yeast, Speciation, Chromosome, Hybrid, Sterility, Anti-recombination, Aneuploidy

## Abstract

Many species are able to hybridize, but the sterility of these hybrids effectively prevents gene flow between the species, reproductively isolating them and allowing them to evolve independently. Yeast hybrids formed by *Saccharomyces cerevisiae* and *Saccharomyces paradoxus* parents are viable and able to grow by mitosis, but they are sexually sterile because most of the gametes they make by meiosis are inviable. The genomes of these two species are so diverged that they cannot recombine properly during meiosis, so they fail to segregate efficiently. Thus most hybrid gametes are inviable because they lack essential chromosomes. Recent work shows that chromosome mis-segregation explains nearly all observed hybrid sterility—genetic incompatibilities have only a small sterilising effect, and there are no significant sterilising incompatibilities in chromosome arrangement or number between the species. It is interesting that chromosomes from these species have diverged so much in sequence without changing in configuration, even though large chromosomal changes occur quite frequently, and sometimes beneficially, in evolving yeast populations.

## Introduction

The unassuming yeast *Saccharomyces cerevisiae* is rather special to us humans for a couple of reasons. The first is that it helps produce many things that we love to eat and drink, most obviously bread, beer and wine, but also chocolate, coffee, cheese, and many others. People have been enjoying many of these products for a long time, so it is interesting to ponder how and why pre-historic humans first captured the fermentative powers of yeast to transform their plain victuals into something more tasty or boozy. The second, not entirely unrelated, reason is that yeast is a wonderful model organism for scientific studies and experiments. In the decades that have passed since Pasteur published his *Études sur la Bière* (Pasteur 1876), since Emile Christian Hansen fathered yeast genetics at the Carlsberg brewery, and since the unlikely phrase “the awesome power of yeast” was first uttered, the molecular workings of this microbe’s innards have been pored over, poked, and published with an intensity in inverse proportion to its size. But these classic studies, critical to the development of modern molecular genetics, typically focused on just a handful of clonal strains that were confined to the lab, providing little insight into their evolutionary origins. Dobzhansky famously stated that “nothing makes sense in biology except in the light of evolution” (Dobzhansky 1973) and indeed many of the current generation of scientists are now illuminating the biology of yeast by going beyond the lab to capture and study large numbers of individuals from natural populations and species (e.g., Peter et al. 2018). These natural isolates have shown us how interesting yeast really is, giving insights into the potential and actual mechanisms of genetic isolation between species.

The species most similar to *S. cerevisiae* is *S. paradoxus. S. paradoxus c*an be found in the same natural environmental samples as *S. cerevisiae* (e.g. Sniegowski et al. 2002), but it is not typically used by humans for fermentation. The two species can mate and form viable hybrids that can divide just fine by asexual mitosis, but that fail miserably at meiosis: 99% or more of their hybrid gametes are inviable (Hunter et al. 1996). Hybrid sterility thus appears to form a barrier to gene flow between these two species, and the causes of hybrid sterility have, therefore, become a major subject of investigation by scientists wishing to understand how the two species came to be. Since sterility greatly decreases the fitness of hybrids, the incompatibilities that cause hybrid sterility presumably evolved in the parental species. If hybridisation was sufficiently rare, its cost could be so low that incompatibilities could evolve by neutral or nearly neutral processes.

We can now precisely quantify the composition of the hybrid sterility barrier between *S. cerevisiae* and *S. paradoxus*. It has long been inferred that some hybrid gametes are inviable simply because they lack at least one chromosome (all chromosomes are essential in yeast). This inference comes from the observation that the rare gametes that do survive hybrid meiosis, about 1% or less of all hybrid gametes produced, typically have two copies of some chromosomes instead of one copy of each, which normal non-hybrid gametes have (Hunter et al. 1996, Greig et al. 2002). This means that the other gametes must be dead for want of these mis-segregated chromosomes. Chromosome mis-segregation was recently quantified in hybrids for the first time using different colour fluorophores, which can be expressed even in inviable gametes, inserted into both homologous copies (one from each species) of each chromosome in a hybrid diploid (Rogers et al. 2018). By examining the fluorescent colours in the inviable gametes produced by this hybrid, we could show that 97% of hybrid gametes lacked at least one essential chromosome.

The reason for this chromosome mis-segregation is anti-recombination. The genomes of *S. cerevisiae* and *S. paradoxus* have diverged so much that they differ at about 12% of their nucleotide positions (Rogers et al. 2018). Because homologous recombination will be rejected by the anti-recombination mechanism unless there is a run of perfectly identical nucleotides, which is very rare when genomes are so diverged, crossing over between chromosomes of the two species is reduced to about 1% of the normal, non-hybrid, rate (Rogers et al. 2018). And because crossing over normally helps chromosomes to segregate accurately during meiosis, mis-segregation is usually the result (Rogers et al. 2018). In this way, each nucleotide difference between the species acts as a tiny incompatibility, preventing local crossing over and contributing incrementally to hybrid sterility. Consistent with this, deletion of the mismatch repair system genes *MSH2* and *PMS1*, which normally prevent recombination between mismatched sequences, increases hybrid fertility, despite also causing an increase in lethal mutation during mitosis (Hunter et al. 1996). Suppressing the expression of *MSH2* and the RecQ helicase *SGS1* specifically during hybrid meiosis increases recombination, improves chromosome segregation, and results in a 70-fold improvement in hybrid fertility (Rogers et al. 2018, Bozdag et al. 2019, preprint 10.1101/755165).

If 97% of hybrid gametes are killed because anti-recombination prevents efficient chromosome segregation, but 99% of hybrid gametes are inviable, what other factors are responsible for killing the remaining fraction? We have recently shown that certain combinations of genes from the two parental species are under-represented in viable hybrid gametes, indicating that they are incompatible and contribute to hybrid sterility (Bozdag et al. 2019, preprint 10.1101/755165). These types of genetic incompatibilities, often called Bateson–Dobzhansky–Muller incompatibilities or “speciation genes”, are widely predicted by evolutionary theory and have been identified as causes of hybrid sterility in other taxa (ex: rice, Long et al. 2008; mice, Mihola et al. 2009; flies, Ting et al. 1998). Consistent with the high proportion of the reproductive barrier between *S. cerevisiae* and *S. paradoxus* that is due to anti-recombination, we find that these incompatible combinations of genes are not completely lethal. They have a relatively small effect on gamete viability, occurring about half as often as expected if they were completely compatible (Bozdag et al. 2019, preprint 10.1101/755165).

Together, anti-recombination and gene incompatibility are sufficient to explain the observed sterility of yeast hybrids, leaving another potential factor—chromosomal incompatibility—conspicuously absent. A difference between species in the number of chromosomes or arrangement of genes on those chromosomes could affect hundreds or thousands of genes, with potentially dramatic effects on hybrid compatibility. For example, a single translocation involving at least one essential gene would reduce hybrid fertility by at least 25% (50% for a reciprocal exchange of chromosome ends containing essential genes). Gain or loss of a chromosome (aneuploidy) as a result of mitotic mis-segregation would mean that hybrids had odd numbers of chromosomes, preventing efficient meiotic segregation, and producing gametes with variable chromosome stoichiometries. From mutation accumulation experiments in haploids, duplications of complete genes are found to occur at a higher rate than base substitutions on a per-cell-division basis (Lynch et al. 2008) and experiments in diploids show similarly high rates of large-scale chromosomal changes (Zhu et al. 2014, Sharp et al. 2018). We would, therefore, expect that species with highly diverged genomes would also differ greatly in their chromosome configuration. However, chromosomal differences are rare between *Saccharomyces* sensu stricto species (Fischer et al. 2000, Kellis et al. 2003). Although the *S. cerevisaie* and *S. paradoxus* strains that we crossed both have 16 pairs of almost perfectly co-linear chromosomes, so all essential genes were arranged on the same number of chromosomes in the same order, the observed hybrid fertility was nevertheless < 1% (Bozdag et al. 2019, preprint 10.1101/755165). Thus, incompatibilities due to differences in chromosome configuration did not contribute anything to hybrid sterility in our between-species cross (Rogers et al. 2018, Bozdag et al. 2019, preprint 10.1101/755165).

How is it then that two species can accumulate about a million small mutations—12% of their entire genomes—yet retain the same gene order and chromosome number? One possibility is that large chromosomal changes reduce fitness so strongly that chromosome configuration is conserved by purifying selection. However, several laboratory studies show that large chromosomal changes can actually be beneficial, at least under stressful conditions. In glucose-limited chemostats, rearrangements and aneuploidies become common after only a few hundred asexual generations (Dunham et al. 2002). The fact that some of the same chromosomal changes appeared independently in different populations, and that many included regions containing genes involved in glucose metabolism, suggests that the rearrangements were beneficial and spread under selection. Rearrangements also appear when cells are starved, putatively because they help cells to survive (Coyle and Kroll 2008). Aneuploidy occurs during adaptation to salt (Dhar et al. 2011), copper (Gerstein et al. 2015), heat stress (Yona et al. 2012, Millet et al. 2015), and as a result of HSP90 inhibition, producing aneuploids that are resistant to various drugs (Chen et al. 2012). Aneuploidy appears to be a common evolutionary adaptation to compensate for the deletion of certain yeast genes (Hughes et al. 2000, Rancati et al. 2008). Major changes in chromosome configuration are also observed in yeast used for fermentation, conferring resistance to high levels of ethanol in maturing sherry (Infante et al. 2003) or to the sulphites found in wine (Perez-Ortin et al. 2002). Ploidy shifts have also been observed in many beer strains (Gallone et al. 2016). Outside of the lab or brewery, chromosomal rearrangement and aneuploidy is found in copper-adapted wild strains isolated from Evolution Canyon in Israel, a location relatively undisturbed by humans (Chang et al. 2013), and in pathogenic strains infecting human patients (Zhu et al. 2016). These examples of chromosomal variation might be attributable to specific unusual environmental stresses, but individuals isolated from normal natural habitats also often carry chromosomal abnormalities. Reciprocal translocations are a common cause of partial reproductive isolation between different *S. cerevisiae* strains (Hou et al. 2014), notably in Chinese primeval forests (Wang et al. 2012). Two isolates of yeast from Brazil were found to be reproductively isolated from other known species and so were initially described as a new species, *S. cariocanus* (Naumov et al. 2000), but are now known to be examples of *S. paradoxus* carrying multiple translocations (Dujon and Louis 2017). Translocations and inversions also appear to be well established within some wild populations of *S. paradoxus* in Canada (Leducq et al. 2016, Eberlein et al. 2019) and aneuploidies have been identified in wild *S. cerevisiae* (Gasch et al. 2016). These many examples, from both experimental and natural populations, of variation in the arrangement of genes on chromosomes and in the number of those chromosomes, are not consistent with chromosomal changes always being deleterious to fitness.

The remarkable conservation of chromosomes across these otherwise diverged species thus presents a paradox. How can their chromosomal configuration be maintained by evolution in the long term when it changes frequently, and apparently beneficially, in the short term? In other words, why would chromosomal configuration be conserved between species when it is often variable within single populations of a species? One possibility is that most modified lineages eventually go extinct because of some long term selective disadvantage that isn’t apparent in the short term. Of course, there is the cost of hybrid sterility itself, but this also applies to the many small mutations that cause chromosome mis-segregation due to anti-recombination. Something seems to be different about large chromosomal changes, which usually prevents them from accumulating in diverging yeast species. Perhaps, because large-scale chromosome changes affect so many genes at once, they affect not only the trait under selection but also other traits affected by all of those other genes. The cost of shifting those other traits may be initially outweighed by the strength of selection for the targeted trait, but later environmental changes can switch the balance, causing extinction of the new chromosomal variant and the long-term preservation of its ancestor. In other words, large chromosomal changes may be adaptive mutations that trap an evolving population in a more specialised, confined, and ultimately ephemeral niche, whilst generalists that retain the ancestral configuration may persist. Such a speculative model would fit the observation of frequent chromosomal changes in adapting populations but long term conservation of chromosomes across species.

There is an alternative to the idea that lineages with changed chromosome configurations become extinct, as they are eventually replaced by their ancestors (or their ancestors’ unchanged offspring). It is possible that individuals carrying a new configuration might themselves produce offspring with another change, one which restores the ancestral arrangement. This is appealing because conservation of the ancestral configuration would not require that some ancestral types persist, despite their short term inferiority, such that they can eventually stage a comeback. New chromosome configurations could become entirely fixed within a population but could later regenerate the old chromosome types again. A population containing chromosomal variants, each affecting multiple traits, could adapt to an unpredictable environmental change. Then, when the environment changes back or when other adaptive mutations optimising the specific trait under selection relieve the stress, the ancestral configuration could be restored, readying the population for the next unpredictable change. Indeed, authors have suggested that this kind of reversible chromosomal change in yeast might itself be a general adaptive strategy to an unpredictably changing environment (Gilchrist and Stelkens 2019, Dunham et al. 2002, Infante et al. 2003, Cox and Bevan 1962). If this inference is correct, we would predict that the rate of production of chromosomal variants would evolve to reflect the probability of environmental fluctuation, as a form of biological bet-hedging (de Jong et al. 2011).

The mechanisms by which chromosome configurations change are consistent with the idea that they could be underlying some kind of phenotypic switch. A mitotic mis-segregation produces cells with both an increased and a decreased chromosome number, so aneuploidy can be reversed by another mis-segregation. In fact, aneuploids appear to have an increased rate of mis-segregation, such that aneuploid populations tend to evolve back to more stable euploid chromosome numbers (Potapova et al. 2013, Zhu et al. 2012). Millet et al. (2015) found that, under heat stress, haploid cells became diploid except for chromosome VIII, which remained in single copy. When returned to normal growth temperatures, euploid diploid colonies arose, carrying a growth advantage (Millet et al. 2015). In sustained heat stress, Yona et al. (2012) found that otherwise-diploid cells carrying an additional copy of chromosome III had increased heat-tolerance, and were initially selected. However, when these aneuploids were allowed to evolve further, other mutations conferred improved heat-tolerance, and the trisomic strains returned to pure diploidy (Yona et al. 2012). In addition, Tan et al. (2013) showed that gain and loss of chr XVI allows a strain to toggle between different colony morphologies. Similarly, chromosomal rearrangements are caused by ectopic recombination, so rearrangements will tend to occur between sites in the genome that share sequence homology. Therefore, a second rearrangement between the same sites can restore the original configuration, potentially enabling a kind of reversible phenotypic switch. Chang et al. (2013) found that chromosome rearrangements providing increased resistance to copper occurred at sites containing Ty transposon insertions. When copper stress was removed, further rearrangement at these Ty sites restored the ancestral chromosome arrangement (Chang et al. 2013). Authors have speculated that Ty sites enabling advantageous reversible rearrangements might be conserved by selection (Dunham et al. 2002), so examining the evolutionary conservation of such sites between populations and between species could provide evidence for the evolution of bet-hedging as well as insight into the environments that past populations were exposed to.

To conclude, we now know that nearly all of the hybrid sterility barrier between two yeast species is due to anti-recombination, with only a small amount due to genetic incompatibilities, and little or none (depending on the specific cross) due to large differences in their chromosomes. Given how often chromosomal variation is observed within yeast populations, it is surprising that yeast chromosomes are so well conserved across highly divergent species. This conservation could be explained if the variation is actually a reversible mechanism underlying a form of stochastic phenotype switch that has evolved to optimise long term fitness in unpredictably fluctuating environments. Investigating this intriguing possibility will require more information about the kinds of habitats yeast live in and, therefore, what kinds of selection pressures might drive their evolution. Many other questions remain open about yeast in their natural habitat. For example, how often they have sex and whether that plays a big role in their fitness. How much gene flow there is between populations and, therefore, the dynamics of how a given rearrangement might change in frequency over time. We don’t even know their full geographic or ecological range. Maybe by knowing the natural habitat and lifestyle of yeast better, we can hope to understand how selection acts on their chromosomes.
